# CSF Markers of Oxidative Stress Are Associated with Brain Atrophy and Iron Accumulation in a 2-Year Longitudinal Cohort of Early MS

**DOI:** 10.3390/ijms241210048

**Published:** 2023-06-12

**Authors:** Andrea Burgetova, Petr Dusek, Tomas Uher, Manuela Vaneckova, Martin Vejrazka, Romana Burgetova, Dana Horakova, Barbora Srpova, Marta Kalousova, Libuse Noskova, Katerina Levova, Jan Krasensky, Lukas Lambert

**Affiliations:** 1 Department of Radiology, First Faculty of Medicine, Charles University and General University Hospital in Prague, 121 08 Prague, Czech Republic; andrea.burgetova@vfn.cz (A.B.); petr.dusek@vfn.cz (P.D.); manuela.vaneckova@vfn.cz (M.V.); romana.burgetova@fnkv.cz (R.B.); jan.krasensky@vfn.cz (J.K.); 2 Department of Neurology, First Faculty of Medicine, Charles University and General University Hospital in Prague, 121 08 Prague, Czech Republic; tomas.uher@vfn.cz (T.U.); dana.horakova@vfn.cz (D.H.); barbora.srpova@vfn.cz (B.S.); 3 Institute of Medical Biochemistry and Laboratory Diagnostics, First Faculty of Medicine, Charles University and General University Hospital in Prague, 121 08 Prague, Czech Republic; martin.vejrazka@lf1.cuni.cz (M.V.); libuse.noskova@vfn.cz (L.N.); katerina.levova@vfn.cz (K.L.); 4 Department of Radiology, Third Faculty of Medicine, Charles University, 100 34 Prague, Czech Republic

**Keywords:** multiple sclerosis, magnetic resonance imaging, cerebrospinal fluid, oxidative stress, iron, susceptibility

## Abstract

In this prospective longitudinal study, we quantified regional brain volume and susceptibility changes during the first two years after the diagnosis of multiple sclerosis (MS) and identified their association with cerebrospinal fluid (CSF) markers at baseline. Seventy patients underwent MRI (T1 and susceptibility weighted images processed to quantitative susceptibility maps, QSM) with neurological examination at the diagnosis and after two years. In CSF obtained at baseline, the levels of oxidative stress, products of lipid peroxidation, and neurofilaments light chain (NfL) were determined. Brain volumetry and QSM were compared with a group of 58 healthy controls. In MS patients, regional atrophy was identified in the striatum, thalamus, and substantia nigra. Magnetic susceptibility increased in the striatum, globus pallidus, and dentate and decreased in the thalamus. Compared to controls, MS patients developed greater atrophy of the thalamus, and a greater increase in susceptibility in the caudate, putamen, globus pallidus and a decrease in the thalamus. Of the multiple calculated correlations, only the decrease in brain parenchymal fraction, total white matter, and thalamic volume in MS patients negatively correlated with increased NfL in CSF. Additionally, negative correlation was found between QSM value in the substantia nigra and peroxiredoxin-2, and QSM value in the dentate and lipid peroxidation levels.

## 1. Introduction

Multiple sclerosis (MS) is a chronic progressive neuroinflammatory demyelinating disease. Its MRI hallmarks are the presence and progression of white and gray matter lesions, atrophy, and iron deposition in deep gray matter [1].

The aberrant immunological response is reflected in the cerebrospinal fluid (CSF). Increased levels of markers of brain tissue degradation, astrocytic activation, oxidative stress, and lipid peroxidation have been identified in the CSF of MS patients, even at the onset of the disease [2,3]. Neurofilament light chain (NfL), which reflects neuro-axonal damage, has been studied as a biomarker of disease activity [4,5]. Increased levels of 8-hydroxy-2′-deoxyguanosine (8-OHdG), peroxiredoxin-2 (PRDX2), and malondialdehyde and hydroxyalkenals (MDA+HAE), as markers of oxidative stress and lipid oxidation, have been identified in treatment naïve MS patients [3,6].

After undergoing brain MRI, lesion load and brain atrophy predict the further development of the disease [1,7]. Iron concentration in the brain, measured by T2* relaxometry or quantitative susceptibility mapping (QSM), increases in the deep gray matter (DGM), particularly in the putamen, globus pallidus, and nucleus caudatus [8,9]. Iron concentration in previously active enhancing lesions shows a marked increase when the blood–brain barrier is restored, which indicates the role of iron homeostasis in the active termination of the inflammatory response [10]. However, iron concentration in the basal ganglia is also correlated with their atrophy. It has been suggested that increased iron concentration may be the result of a loss of regional tissue volume, leading to a higher iron density rather than frank iron influx [11].

To obtain a better understanding of the disease’s natural course and its management, variables that predict disease progression in the early stages of the disease should be investigated in longitudinal research [12,13]. Currently, NfL is the most studied biochemical marker in MS and it has been convincingly shown that higher baseline serum and CSF NfL levels predict brain parenchymal volume loss during the follow-up period [13,14]. To the best of our knowledge, no studies examined the predictive power of oxidative stress markers on the development of MRI metrics in MS.

The aim of this study was to quantify (1) brain volume and (2) magnetic susceptibility changes in DGM structures at two years after MS diagnosis, and (3) to find baseline predictors of these changes, with particular focus on NfL and CSF markers related to oxidative stress.

## 2. Results

**Figure 1:**
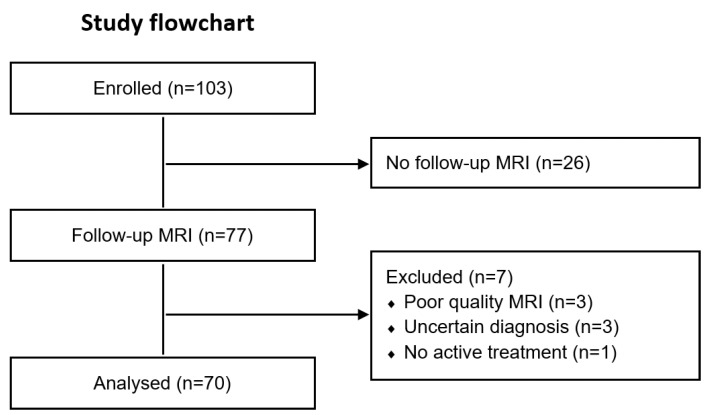
Study flowchart.

From the original 103 MS patients enrolled at baseline, 26 patients did not undergo a follow-up MRI after 2 years (retention rate 75%). Additionally, we excluded 3 patients due to poor-quality MRI, 3 patients for uncertain MS diagnosis (suspected neuromyelitis optica spectrum disorder or overlap), and 1 patient who did not receive disease-modifying treatment, resulting in the inclusion of 70 patients for the final longitudinal analysis (Figure 1).

The patients underwent initial MRI between August 2017 and January 2020, and follow-up MRI between April 2020 and February 2022. The time between baseline and follow-up MRI, representing approximate disease duration, was 25.1 months (IQR 24.4 to 26.2 months). There were 48 females and 22 males, aged 31 (IQR 26–41) years (Table 1). The type of disease-modifying treatment is shown in Supplementary Table S1. There were 58 healthy controls with baseline and follow-up MRI [22 (38%) males, age 38 (IQR 30–47) years].

In MS patients between the baseline and follow-up MRI, brain parenchymal fraction decreased, while significant volume loss was found for the total white and gray matter, caudate, thalamus, substantia nigra, and putamen (Table 2). At the same time, mean bulk magnetic susceptibility significantly increased in the caudate, GP, putamen, and dentate, and decreased in the thalamus. After adjustment to atrophy, magnetic susceptibility changes remained significant in all nuclei other than the dentate (Table 2). MS patients received 15 (IQR 13–20) mL of 0.5 M-equivalent Gd-based contrast material. The volume of contrast material was not correlated with the susceptibility change in the globus pallidus (*p* = 0.26) or in the dentate (*p* = 0.95). There were 19 (27%) patients, with higher EDSS seen at two years compared to the baseline. Binary multivariable analysis (baseline quantitative MRI and biochemical values) yielded no significant predictive model for understanding EDSS change.

The pattern of longitudinal changes in regional volumes and susceptibilities in HC was similar to that of the MS group. Compared to HC, MS patients developed greater atrophy of the thalamus and total white matter, and a greater increase in susceptibility in the caudate, putamen, globus pallidus and greater decrease in susceptibility in the thalamus (Supplementary Table S2).

**Figure 2:**
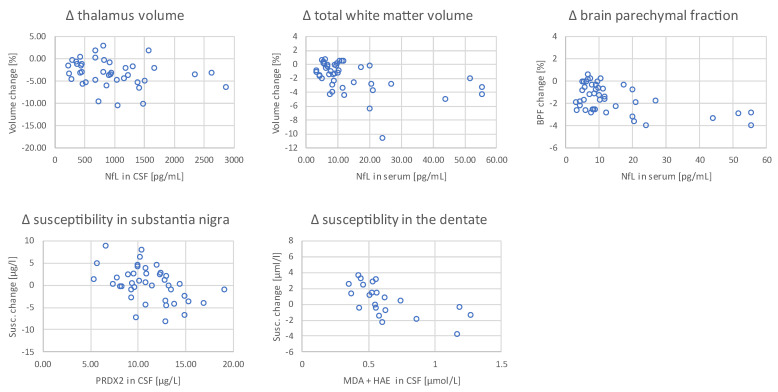
Correlation of changes in thalamic and total white matter volume, brain parenchymal fraction (BPF) with neurofilament light chain levels (NfL) in cerebrospinal fluid (CSF) and serum (above). Correlation of changes in susceptibility in substantia nigra and the dentate with peroxiredoxin-2 (PRDX2) and malondialdehyde + hydroxyalkenals (MDA + HAE) in CSF (below).

When the magnitude of these changes (volume, susceptibility) in MS patients was correlated with the level of biochemical parameters at baseline, a decrease in brain parenchymal fraction as well as white matter and thalamic volume loss were positively associated with higher NfL in CSF and serum (Table 3, Figure 2). Lower CSF PRDX2 levels at baseline were associated with a greater increase in magnetic susceptibility in the substantia nigra. Lower CSF MDA + HAE levels at baseline were associated with a greater increase in magnetic susceptibility in the dentate (Table 3). 

## 3. Discussion

This longitudinal study showed excessive volume loss of thalamic nuclei and white matter and excessive iron accumulation in the striatum and globus pallidus in early MS patients compared to healthy controls. None of these abnormalities were related to CSF oxidative stress markers, while white matter and thalamic volume loss were correlated with increased NfL in CSF and serum at baseline. Despite not being significantly altered at the group level, a greater increase in magnetic susceptibility in the substantia nigra and dentate nucleus correlated with lower baseline levels of PRDX2 and lipid peroxidation products in MS patients. 

Consistent with previous studies, our study observed global gray and white matter atrophy, together with thalamic and striatal volume loss in MS patients between baseline and 2 years’ follow-up [15]. Of these, thalamic and white matter volume loss were significantly greater than those seen in physiological aging. These neurodegenerative components of MS were previously shown to progress throughout the disease duration and to possess a strong predictive potential for disability and cognitive impairment [16,17].

The only predictor of atrophy in our current study was NfL [18,19]. NfL levels in CSF and serum are established as an early indicator of disease activity, consistently predict future brain tissue loss, and improve the ability to identify patients at higher risk of future disease activity [4,18,20]. More specifically, baseline NfL levels in our study correlated with thalamic atrophy, as has already been reported previously [14]. The thalamus is one of those structures where atrophy is first measurable in MS patients. It has a particularly strong clinical correlation and enables clinicians to predict the progression of disability [15,21]. An association can thus be expected between thalamic atrophy and NfL levels. A study by Jakimovsky et al. reported an association between higher Nfl levels and lower rates of thalamic perfusion. Both perfusion changes and atrophy, occurring as a result of neurodegeneration, may describe the overlapping pathophysiological mechanisms of MS [22].

In our MS patients, magnetic susceptibility generally increased in DGM structures. This was notable in the caudate, putamen, and GP, and it exceeded the increase observed in physiological aging. This observation supports previous reports of the increased magnetic susceptibility or R2* relaxation rate in the basal ganglia of MS patients interpreted as iron accumulation [23,24]. A magnetic susceptibility increase was apparent even after correction for atrophy, suggesting that the higher regional iron density could not be explained by atrophy alone [11]. It was also not related to the amount of Gadolinium contrast material which could accumulate in the globus pallidus and dentate nucleus. Most likely, increased magnetic susceptibility was caused by the combined effect of an influx of paramagnetic iron due to neurodegeneration and the loss of diamagnetic myelin [25]. Histopathological studies in MS patients showed that iron is primarily stored within oligodendrocytes and myelin fibers and is released upon demyelination [26,27]. In contrast to basal ganglia, decreased susceptibility was found in the thalamus in MS compared to HC, as has already been reported in previous studies [23,24]. The accelerated decrease in thalamic susceptibility might have been due to the involvement of chronic microglia activation in iron depletion from oligodendrocytes [28].

Contrary to our hypothesis, CSF markers of oxidative stress did not predict volume loss or iron accumulation in the major brain structures affected by MS. Oxidative stress has been implicated as a mediator of demyelination and axonal damage in MS and this is reflected by the upregulation of anti-oxidative enzymes, such as glutathione peroxidase or peroxiredoxins, or by the increase in lipid peroxidation markers related to disease severity [6,29,30,31]. In our previous cross-sectional study on this cohort, we observed increased CSF levels of PRDX2 that correlated with thalamic atrophy in MS patients. This highlighted the active role of anti-oxidative cytoprotective enzymes in the termination of the inflammatory reaction [3,32]. The lack of association between PRDX2 and other markers of oxidative stress with the degree of volume loss in the longitudinal study may be theoretically related to the positive effect of anti-inflammatory treatment on oxidative stress in MS [30,33].

Significant associations between oxidative stress markers and magnetic susceptibility were found for substantia nigra and dentate nucleus, structures that have been only marginally studied in MS. Interestingly, several susceptibility networks of brain regions with the independent regulation of iron homeostasis during aging were recently described and shown to be different for healthy and MS individuals [34]. Our finding of a selective association between oxidative stress markers and magnetic susceptibility increase, discovered only in a subset of DGM, supports the theory of several independent mechanisms of iron accumulation in different brain regions. PRDX2 negatively correlated with iron accumulation in the substantia nigra; however, this did not significantly increase at the group level after a period of two years in our study. Nevertheless, in another study, iron accumulation in the substantia nigra was found to be accelerated, suggesting that the substantia nigra may accumulate iron in a subgroup of MS patients with low CSF PRDX2 levels [35]. PRDX2 is a highly reactive peroxidase with a cytoprotective function. In the brain, it is predominantly expressed in astrocytes in the white matter, and its highest concentrations are found at the periphery of demyelinating lesions [36]. The mechanism of the protective effect of PRDX2 against iron accumulation in the substantia nigra is unclear. It is important to note that the role of the substantia nigra in the pathogenesis of MS may be underestimated; a recent study showed that fatigue is specifically linked with microglial activation in the substantia nigra [37]. We can also only speculate why only PRDX2 of all examined oxidative stress markers was associated with substantia nigra iron content. Peroxiredoxin acts as an important system that scavenges reactive oxygen species and protects the cell from oxidative damage. In this way, it rapidly attenuates the production of other markers of oxidative tissue damage. Thus, PRDX2 can be considered to be a more sensitive marker of reactive oxygen species production in many situations [38,39].

In the dentate, magnetic susceptibility negatively correlated with baseline MDA+HAE levels. The structural abnormalities of the dentate nucleus, consisting of reduced afferent synapses and astroglial reaction in MS, have been previously shown [40]. MDA and HAE are markers of lipid peroxidation in sensitive brain tissue with high oxygen consumption and a high concentration of polyunsaturated fatty acids and serve as reliable markers of oxidative stress-mediated lipid peroxidation [41]. Iron-dependent lipid peroxidation by reactive oxygen species has been described as ferroptosis [42]. Lipid peroxidation is also implicated in demyelination [43]. Therefore, the inverse relationship between the increase in magnetic susceptibility in the dentate, which may result from iron accumulation or demyelination, and baseline MDA+HAE levels is counterintuitive [31]. Further studies are needed to confirm this finding and to better understand the relations among lipid peroxidation, iron accumulation, myelin damage and repair processes in MS.

Our study has several limitations. First, we did not investigate the dynamics of CSF markers at follow-up examination on treatment due to patients’ unwillingness to undergo a second lumbar puncture. Second, the relatively high number of patients lost to follow-up (25%) decreased the power of the study. Third, two years is a relatively short time to assess disease progression and no predictors of disability could be identified since no significant change of EDSS was observed during the follow-up period. Fourth, MS patients were younger than the controls. This bias is, however, mitigated by the longitudinal design. Finally, the time between baseline and follow-up MRI was longer in HC than in MS patients.

## 4. Materials and Methods

This study (ClinicalTrials.gov ID: NCT03706118) was approved by the Ethics Committee of the General University Hospital in Prague (ID1018/17, 52/17). It was carried out in accordance with the Declaration of Helsinki and all subjects signed informed consent. 

### 4.1. Study Participants

The study group comprised treatment-naive relapse-remitting MS patients de novo diagnosed between August 2017 and January 2020 who underwent neurological examination, including via expanded disability status scale (EDSS), brain MRI, and CSF sampling at baseline as well as neurological examination and brain MRI 2 years later [44,45]. The inclusion criteria were aged ≥18 years and diagnosis of MS according to the 2017 McDonald criteria. We excluded patients with other diseases affecting the brain and pregnant women.

All patients received disease-modifying treatment (Supplementary Table S1). The interval between corticosteroid treatment and MRI was longer than 30 days in all patients. A total volume of 0.5M equivalent of macrocyclic gadolinium (Gd)-based contrast material was recorded between MRI at baseline and at follow-up.

Healthy controls (HC) were recruited from the general community [3,45]. The controls were free of neurologic or other medical disorders affecting the brain and had a normal neurological examination. HC underwent a brain MRI at baseline and 4 years later.

The imaging protocol, image processing, and CSF assays have been described in detail in our previous work [3].

### 4.2. Imaging Protocol

MRI examinations were performed at baseline and follow-up after two years using the same scanner and protocol as previously described [45]. Briefly, magnetization-prepared rapid gradient-echo (MPRAGE, TE: 2.96 ms, TI 900 ms TR: 2300 ms, spatial resolution: 1 × 1 × 1 mm^3^), gradient-echo (GRE, 6 TEs: 4.5–29.5 ms, evenly spaced, TR: 33 ms, spatial resolution: 0.94 × 0.94 × 0.94 mm^3^), and fluid-attenuated inversion recovery (FLAIR, TE 397 ms, TI 1800 ms, TR 5000 ms, spatial resolution 1 × 1 × 1 mm^3^) pulse sequences were acquired on a 3T MRI scanner (Siemens Skyra 3T, Siemens Healthcare, Erlangen, Germany) with a 20-channel head coil [3].

### 4.3. Image Processing

GRE images were processed to generate quantitative susceptibility maps (QSM) using a multi-scale dipole inversion-based pipeline for coil-combined, multi-gradient echo data in QSMbox (https://gitlab.com/acostaj/QSMbox, accessed on 20 June 2022) [46]. The statistical Parametric Mapping (SPM12, version 7771; http://www.fil.ion.ucl.ac.uk/spm, accessed on 1 February 2020) and Computational Anatomy Toolbox software (CAT12, version 12.8.1; www.neuro.uni-jena.de/cat12/, accessed on 20 October 2020), running under Matlab v. 2022a (The Math Works, Inc., Natick, MA, USA), were used for the processing of T1-weighted MPRAGE images and coregistration. First, individual T1-weighted images from both time points were denoised, underwent bias-field correction, and realigned with a rigid-body registration using the longitudinal registration pipeline in CAT12. Next, FLAIR images were registered to the corresponding T1-weighted images and white matter lesions were segmented via the lesion prediction algorithm as implemented in the LST toolbox version 3.0.0 (www.statistical-modelling.de/lst.html, accessed on 1 June 2022) for SPM; the resulting lesion map was used for lesion filling applied to T1-weighted images [47]. T1-weighted images were then segmented using CAT12 to obtain total volumes of gray and white matter and brain parenchymal fraction (BPF). Subsequently, the first-echo GRE magnitude image was registered to the corresponding T1-weighted image and the registration matrix was applied to QSM using nearest neighbor interpolation to align QSM to the space of T1-weighted images. T1-weighted images were skull-stripped by multiplication with an SPM12-based brain binary mask calculated with QSMbox software.

Finally, co-registered skull-stripped QSM and T1-weighted images entered a fully automated multi-atlas segmentation pipeline using dual contrast for the delineation of DGM nuclei implemented in a cloud-based platform (www.mricloud.org, accessed on 30 October 2022) [48]. Quality assessment was performed for each lesion and deep gray matter segmentation by a trained researcher to ensure the correctness of the segmentation. The volumes of the caudate, globus pallidus, putamen, thalamus, subthalamic nucleus, red nucleus, and dentate nucleus were reported as the sum of bilateral structures. Susceptibility values were referenced to the whole-brain mean susceptibility to avoid potential bias from disease-related susceptibility changes in specific anatomic areas or from the manual delineation of reference regions [49]. To account for iron concentration increases due to atrophy, we also calculated the susceptibility mass of each DGM region by multiplying its volume by the mean bulk susceptibility [11,50]. 

### 4.4. CSF and Serum Assays

In MS patients, at baseline, CSF was collected using an atraumatic needle to deposit the material from the L5-S1, L4-5, or L3-4 interspace together with a venous blood sample into polypropylene test tubes. Approximately 1 mL of CSF was used for routine analysis of albumin and total CSF protein levels, white blood cell count, IgG index, oligoclonal bands, and CSF/serum albumin ratio (Table 1). Sera and CSF were separated within 60 min after collection using centrifugation at 3000 rpm for 10 min. Blood and CSF were centrifuged at room temperature and 4 °C, respectively. After centrifugation, CSF and sera were immediately aliquoted and frozen at −80 °C until further biochemical analysis, i.e., the samples passed through only one freeze–thaw cycle.

The levels of 8-iso prostaglandin F_2α_ (8-isoPG, 8-isoprostane), neutrophil gelatinase-associated lipocalin (NGAL, lipocalin-2), peroxiredoxin-2 (PRDX2), 8-hydroxy-2′-deoxyguanosine (8-OHdG), and of the products of lipid peroxidation (malondialdehyde and 4-hydroxyalkenals, MDA + HAE) were determined using ELISA and colorimetric methods, as previously described [3]. Neurofilament levels in CSF (CSF-NfL) were measured by enzyme-linked immunosorbent assays (ELISA) using the NF-light ELISA kit (UmanDiagnostics AB, Umea, Sweden). Serum NfL concentration was measured using a sensitive immunoassay on the Simoa platform (Quanterix, Billerica, MA, USA).

### 4.5. Statistical Analysis

Statistical analysis was performed in SPSS 19 (IBM, Armonk, NY, USA) and R (R Core Team, Vienna, Austria). Variables were compared using paired the Wilcoxon test and Mann–Whitney test. Gender- and age-adjusted Spearman’s rank coefficients among biochemical markers and deep gray matter volumes and susceptibilities were calculated (pcor function in R). For adjustment to determine family-wise error, the Bonferroni correction was used (p.adjust function in R). Binary multivariable analysis was performed using a forward model. In order to compare the quantitative analysis of brain MRI between MS patients and healthy controls, changes were adjusted to the interval between baseline and follow-up MRI. A *p* value < 0.05 was considered significant.

## 5. Conclusions

Serum and CSF NfL levels, as markers of axonal injury, predict early decreases in brain parenchymal fraction. This primarily occurs due to thalamic atrophy and white matter volume loss. CSF markers of oxidative stress are not associated with tissue loss or iron dysregulation in the striatum, globus pallidus, and thalamus, arguing against oxidative stress playing an eminent role in early disease progression in treated MS patients. Decreased CSF PRDX2 levels correlate with iron accumulation in the substantia nigra and decreased CSF MDA+HAE levels correlate with iron accumulation in dentate nucleus. Identification of novel biomarkers of disease activity that predict disease progression may contribute to a better understanding of disease pathophysiology and has the potential to improve treatment strategies for MS patients. These findings indicate different underlying mechanisms of iron accumulation in the substantia nigra and dentate nucleus, possibly related to oxidative stress.

## Figures and Tables

**Table 1:** Demographic and biochemical data.

	MS Patients	Healthy Controls	*p*
Number of subjects	70	58	
Sex [male/female]	22 (31%)	22 (38%)	0.46
Age [years]	31 (IQR 26-41)	38 (IQR 30–47)	0.0020
Time between first and second MRI [years]	2.1 (IQR 2.0–2.2)	4.1 (IQR 4.0–4.2)	<0.0001
Freezer storage time of samples [years]	1.5 (IQR 1.0–2.0)	-	-
CSF white blood cells/mm^3^ [n]	17 (IQR 7–37)	-	-
CSF total protein [g/L]	0.32 (IQR 0.24–0.43)	-	-
CSF albumin [mg/L]	204.0 (IQR 152.5–267.5)	-	-
CSF IgG index [a.u.]	0.9 (IQR 0.6–1.4)	-	-
CSF oligoclonal bands [n]	15 (IQR 10–23)	-	-
CSF/serum albumin ratio [a.u.]	4.6 (IQR 3.3–6.1)	-	-

**Table 2:** Changes in volume and susceptibility in deep gray matter structures and whole brain in the first two years after the diagnosis of MS.

	Baseline		Follow-Up 2 Years		Change	
	Mean	IQR	Mean	IQR	%	*p* Value
**Volume** [cm^3^]						
Caudate	7.79	7.27 to 8.31	7.65	7.26 to 8.16	−1.8	**<0.0001**
Putamen	8.72	8.11 to 9.34	8.48	7.93 to 9.21	−2.8	**0.0038**
Globus pallidus	4.24	4.03 to 4.57	4.32	4.01 to 4.62	1.9	0.428
Thalamus	11.5	10.6 to 12.2	10.9	10.4 to 11.9	−5.2	**<0.0001**
Subthalamic nucleus	0.339	0.31 to 0.38	0.34	0.31 to 0.38	0.3	0.315
Substantia nigra	1.33	1.2 to 1.45	1.27	1.21 to 1.38	−4.5	**0.0070**
Red nucleus	0.615	0.58 to 0.65	0.61	0.57 to 0.65	−0.8	0.392
Dentate	1.85	1.64 to 2.14	1.87	1.63 to 2.08	1.1	0.398
Total grey matter	651	614 to 693	643	615 to 673	−1.2	**<0.0001**
Total white matter	508	476 to 546	501	465 to 540	−1.4	**<0.0001**
Brain parenchymal fraction [%]	0.81	0.79 to 0.82	0.80	0.78 to 0.81	−1.2	**<0.0001**
**Susceptibility** [ppb]		-				
Caudate	20.5	17.9 to 24.8	21.7	19 to 25.6	5.9	**<0.0001**
Putamen	20.3	15.7 to 23.3	21.2	17.1 to 24.8	4.4	**<0.0001**
Globus pallidus	55.7	51.6 to 60.7	56.2	51.9 to 61.9	0.9	**0.0012**
Thalamus	−2.15	−3.23 to −0.77	−3.00	−3.89 to −1.42	−39.5	**<0.0001**
Subthalamic nucleus	38.7	34.2 to 43.5	38.7	35.3 to 44.2	0.0	0.497
Substantia nigra	49.7	45.1 to 55.2	49.4	44.9 to 55.7	−0.6	0.249
Red nucleus	36.5	30.1 to 42.2	37.8	28.6 to 41.7	3.6	0.712
Dentate	33	28.1 to 39.3	33.1	30 to 40.4	0.3	**0.0026**
**Susceptibility mass** [ppb·cm^3^] ^1)^						
Caudate	161	139 to 191	165	144 to 198	2.5	**0.0085**
Putamen	173	134 to 205	179	148 to 215	3.5	**<0.0001**
Globus pallidus	237	211 to 274	241	215 to 284	1.7	**0.0014**
Thalamus	10.4	−7.82 to 28.8	2.5	−13.3 to 27.0	−76.0	**0.0003**
Subthalamic nucleus	13.4	10.8 to 16.1	13.3	11.3 to 15.9	−0.7	0.635
Substantia nigra	64.7	56.4 to 76.7	64.1	54.9 to 74.2	−0.9	0.361
Red nucleus	22.7	17.9 to 26.7	23.3	17.3 to 26.1	2.6	0.409
Dentate	59	48.9 to 84.3	61.4	50.1 to 84.5	4.1	0.106
**Lesion load**		-				
Lesion load [cm^3^]	0.414	0.140 to 1.040	0.327	0.125 to 0.856	−21.0	0.0553
Lesion count	5	2 to 11.8	5	3 to 10	-	0.666
**EDSS**						
EDSS	2	1.5–2	1.5	1.5–2	-	0.911

EDSS: expanded disability status scale; IQR: interquartile range. ^1)^ Calculated as uncorrected susceptibility × volume. *P*-values in boldface indicate statistical significance.

**Table 3:** Spearmann’s rho correlation coefficients among quantitative changes on MRI in the first two years and CSF and serum markers at baseline. Significant *p*-values (<0.05) are in red.

	8-OHDG		8-IsoPG		NGAL		PRDX2		MDA + HAE		NfL in CSF		NfL in Serum	
	rho	*p*	rho	*p*	rho	*p*	rho	*p*	rho	*p*	rho	*p*	rho	*p*
**Δ Volume** [baseline—follow-up; cm^3^]														
Caudate	0.051	*0.757*	0.174	*0.289*	−0.086	*0.603*	0.196	*0.231*	0.098	*0.681*	0.086	*0.587*	0.072	*0.640*
Putamen	0.091	*0.580*	−0.076	*0.644*	−0.099	*0.548*	0.204	*0.213*	−0.080	*0.738*	−0.005	*0.975*	−0.099	*0.517*
Globus pallidus	−0.190	*0.247*	0.199	*0.226*	0.190	*0.247*	0.038	*0.819*	0.330	*0.155*	0.199	*0.206*	0.184	*0.225*
Thalamus	0.055	*0.741*	−0.161	*0.327*	−0.046	*0.781*	−0.054	*0.746*	−0.075	*0.752*	−0.411	*** 0.007 ***	−0.371	***0.012***
Subthalamic nucleus	−0.230	*0.159*	−0.143	*0.385*	0.121	*0.462*	−0.260	*0.110*	−0.374	*0.105*	0.091	*0.565*	0.173	*0.255*
Substantia nigra	−0.240	*0.141*	−0.097	*0.558*	0.054	*0.742*	−0.059	*0.720*	−0.114	*0.633*	−0.158	*0.318*	−0.137	*0.369*
Red nucleus	−0.123	*0.456*	0.132	*0.424*	0.193	*0.238*	−0.129	*0.435*	−0.255	*0.278*	−0.054	*0.734*	0.077	*0.613*
Dentate	0.132	*0.423*	−0.084	*0.612*	0.048	*0.774*	−0.154	*0.349*	0.049	*0.837*	0.208	*0.187*	0.005	*0.973*
Total grey matter	−0.076	*0.644*	−0.095	*0.565*	0.061	*0.712*	0.185	*0.259*	−0.293	*0.211*	−0.001	*0.995*	−0.052	*0.734*
Total white matter	−0.082	*0.621*	−0.271	*0.095*	0.112	*0.497*	0.011	*0.949*	0.107	*0.654*	−0.399	***0.009***	−0.456	*** 0.002 ***
Brain parenchymal fraction	−0.066	*0.688*	−0.222	*0.175*	0.079	*0.634*	0.196	*0.233*	−0.109	*0.647*	−0.308	***0.047***	−0.355	***0.017***
**Δ Susceptibility** [baseline—follow-up; ppb] ^1)^														
Caudate	−0.058	*0.724*	0.191	*0.245*	0.009	*0.957*	−0.139	*0.399*	0.077	*0.747*	−0.110	*0.489*	−0.064	*0.678*
Putamen	−0.099	*0.547*	0.157	*0.340*	−0.150	*0.361*	0.111	*0.502*	−0.096	*0.687*	−0.085	*0.593*	−0.060	*0.696*
Globus pallidus	−0.065	*0.692*	−0.223	*0.172*	−0.125	*0.450*	−0.170	*0.301*	−0.102	*0.669*	0.012	*0.938*	−0.152	*0.318*
Thalamus	−0.281	*0.084*	0.136	*0.408*	−0.132	*0.423*	0.145	*0.378*	0.127	*0.592*	−0.017	*0.917*	−0.061	*0.689*
Subthalamic nucleus	−0.056	*0.734*	0.049	*0.768*	−0.020	*0.902*	0.059	*0.719*	−0.014	*0.953*	0.158	*0.316*	−0.139	*0.362*
Substantia nigra	0.138	*0.401*	−0.308	*0.056*	−0.116	*0.481*	−0.407	***0.010***	−0.088	*0.713*	−0.089	*0.576*	−0.180	*0.237*
Red nucleus	−0.048	*0.771*	0.006	*0.970*	−0.162	*0.324*	0.124	*0.453*	0.369	*0.110*	−0.255	*0.104*	−0.195	*0.198*
Dentate	−0.075	*0.648*	−0.315	*0.051*	−0.159	*0.334*	−0.266	*0.102*	−0.559	***0.010***	−0.155	*0.326*	−0.156	*0.306*
**Δ Susceptibility mass** [baseline—follow-up; ppb·cm^3^] ^2)^														
Caudate	−0.031	*0.850*	0.225	*0.169*	0.015	*0.926*	−0.030	*0.855*	0.168	*0.479*	0.009	*0.952*	0.006	*0.971*
Putamen	0.024	*0.883*	0.098	*0.551*	−0.162	*0.324*	0.190	*0.248*	−0.103	*0.666*	−0.138	*0.382*	−0.168	*0.269*
Globus pallidus	−0.179	*0.275*	0.052	*0.755*	0.110	*0.506*	−0.116	*0.484*	0.255	*0.277*	0.152	*0.338*	0.091	*0.554*
Thalamus	−0.230	*0.159*	0.135	*0.412*	−0.081	*0.622*	0.173	*0.292*	0.147	*0.536*	0.103	*0.516*	−0.066	*0.665*
Subthalamic nucleus	−0.224	*0.170*	−0.111	*0.502*	0.039	*0.814*	−0.205	*0.211*	−0.403	*0.078*	0.092	*0.561*	−0.039	*0.799*
Substantia nigra	−0.089	*0.590*	−0.198	*0.228*	−0.026	*0.876*	−0.307	*0.057*	−0.202	*0.392*	−0.181	*0.250*	−0.183	*0.228*
Red nucleus	−0.020	*0.902*	0.097	*0.557*	0.029	*0.862*	0.020	*0.903*	0.082	*0.731*	−0.305	***0.049***	−0.179	*0.240*
Dentate	0.022	*0.892*	−0.318	***0.049***	−0.109	*0.510*	−0.331	***0.040***	−0.451	***0.046***	0.012	*0.940*	−0.048	*0.754*

8-OHdG: 8-hydroxy-2′-deoxyguanosine; 8-IsoPG: 8-iso prostaglandin F_2α_; NGAL: neutrophil gelatinase-associated lipocalin; PRDX2: peroxiredoxin-2; MDA + HAE: malondialdehyde and 4-hydroxyalkenals; NfL CSF: neurofilament light chain in CSF; NfL serum: neurofilament light chain in serum. *P*-values in boldface indicate statistical significance. Underlined *p* values remain significant (*p* < 0.05) after Bonferroni correction. ^1)^ Corrected for whole brain susceptibility. ^2)^ Calculated as a product of susceptibility and raw regional volume.

## Data Availability

The data presented in this study are available in the article or supplementary material.

## Supplementary Materials

The supporting information can be downloaded at: https://www.mdpi.com/article/10.3390/ijms241210048/s1.
