# Supplementary material for: CSF Markers of Oxidative Stress Are Associated with Brain Atrophy and Iron Accumulation in a 2-Year Longitudinal Cohort of Early MS
Source: Int J Mol Sci. 2023 Jun 12;24(12):10048. doi: 10.3390/ijms241210048 (PMC10298232; doi:10.3390/ijms241210048)
Supplement: Supplementary file 1 [file ijms-24-10048-s001.zip › ijms-2392752-supplementary.pdf]

Suppl. Table S1. Disease modifying treatment in RR MS patients.

Disease Modifying Treatment	Number of Patients
Aubagio	8
Avonex	1
Betaferon	7
no DMT	1
Copaxone [40]	9
Gilenya	5
Mavenclad_doba	6
Ocrevus_doba	5
Plegridy	16
Rebif [22]	1
Rebif [44]	7
Tecfidera	2
Tysabri	2

DMT: disease modifying treatment

Suppl. Table S2. Comparison of changes in volume and susceptibility in deep gray matter structures and whole brain between MS patients and healthy controls per one year.

	MS patients		Healthy controls		
	**Change**	IQR	**change**	IQR	***p***
**Volume** [%]					
Caudate	-0.82	-2.13 to 0.05	-0.4	-0.79 to 0	0.069
Putamen	-0.62	-2.72 to 0.6	-0.56	-1.04 to -0.16	0.71
Globus pallidus	-0.01	-1.1 to 1.64	0.31	-0.09 to 1	0.26
Thalamus	-1.52	-2.42 to -0.41	-0.46	-0.79 to -0.12	**<0.0001**
Subthalamic nucleus	-0.49	-4.18 to 3.15	0.07	-1.19 to 2.3	0.14
Substantia nigra	-1.11	-3.98 to 1.15	-0.25	-1.5 to 0.53	0.16
Red nucleus	-0.52	-3.31 to 2.27	0.01	-1.28 to 0.96	0.18
Dentate	-0.21	-1.7 to 1.21	0.01	-0.67 to 1.15	0.17
Total grey matter	-0.59	-1.27 to -0.06	-0.38	-0.65 to -0.19	0.075
Total white matter	-0.61	-1.31 to -0.11	-0.03	-0.21 to 0.14	**<0.0001**
Brain parenchymal fraction [%]	-0.59	-1.24 to -0.16	-0.22	-0.37 to -0.09	**0.0004**
**Susceptibility** [ppb]					
Caudate	0.53	-0.15 to 0.98	0.19	-0.06 to 0.4	**0.032**
Putamen	0.62	0.09 to 1.11	0.33	0.08 to 0.64	**0.018**
Globus pallidus	0.29	-0.24 to 1.2	0.04	-0.34 to 0.46	**0.027**
Thalamus	-0.25	-0.58 to 0.02	-0.09	-0.22 to 0.05	**0.0071**
Subthalamic nucleus	0.04	-0.92 to 1.14	0.12	-0.69 to 0.68	0.77
Substantia nigra	0.26	-0.96 to 1.35	0.1	-0.62 to 0.61	0.31
Red nucleus	-0.08	-1.04 to 0.83	0.24	-0.18 to 0.8	0.15
Dentate	0.34	-0.22 to 1.07	0.17	-0.08 to 0.54	0.35
**Susceptibility mass** [ppb·cm^3] [1)]					
Caudate	2.22	-1.84 to 6.46	0.79	-1.36 to 2.75	0.16
Putamen	3.94	-0.66 to 7.35	1.88	-0.69 to 4.64	0.066
Globus pallidus	2.91	-1.64 to 6.79	1.13	-1.03 to 4.71	0.17
Thalamus	-2.77	-6.06 to 0.57	-1.02	-2.04 to 0.58	**0.0094**
Subthalamic nucleus	0.02	-0.55 to 0.46	0.05	-0.29 to 0.48	0.35
Substantia nigra	-0.74	-2.64 to 2.36	0.44	-1.24 to 1.77	0.21
Red nucleus	-0.29	-0.97 to 0.79	0.25	-0.25 to 0.79	**0.012**
Dentate	0.61	-1.1 to 2.35	1.00	-0.26 to 2.29	0.17

[1)] Calculated as uncorrected susceptibility × volume. *P*-values in boldface indicate statistical significance.
